# School Lunch Programs and Nutritional Education Improve Knowledge, Attitudes, and Practices and Reduce the Prevalence of Anemia: A Pre-Post Intervention Study in an Indonesian Islamic Boarding School

**DOI:** 10.3390/nu15041055

**Published:** 2023-02-20

**Authors:** Rimbawan Rimbawan, Reisi Nurdiani, Purnawati Hustina Rachman, Yuka Kawamata, Yoshizu Nozawa

**Affiliations:** 1 Department of Community Nutrition, Faculty of Human Ecology, IPB University, Bogor 16680, West Java, Indonesia; 2 Institute of Food Sciences and Technologies, Ajinomoto Co., Inc., Kawasaki City 210-8681, Kanagawa, Japan

**Keywords:** anemia, attitudes, diet, knowledge, practices, nutrition, school lunch program

## Abstract

Indonesians face serious health issues that arise from malnutrition, particularly in children who are under unfavorable dietary environments. The present study established a school meal program consisting of dietary and educational interventions and evaluated its impact on promoting continuous improvement in dietary behavior among junior and senior high school students in Indonesia. A total of 319 students belonging to an Islamic Boarding School participated in the pre-post intervention study for 9 months. All participants were assessed based on their Knowledge, Attitude, and Practice (KAP). A subgroup of 115 participants who were anemic and underweight was examined for dietary intake, nutrition status, and hemoglobin level. The KAP test scores for both nutrition and hygiene showed a significant increase for all students and the undernutrition group post-intervention. Protein, iron, and vitamin C intake significantly improved. Although there were no significant improvements in nutrition status, there was a significant increase in the hemoglobin level and a reduction in the prevalence of anemia from 42.6% to 21.7%. Thus, school meal program that combines dietary and educational interventions may effectively improve anemia in undernourished students as well as enhance the knowledge, attitudes, and practices related to health, nutrition, and hygiene in junior and senior high school students.

## 1. Introduction

Non-communicable diseases, including lifestyle diseases due to overnutrition, are on the rise as a result of economic development, particularly in Southeast Asia and South America, while health issues related to malnutrition, such as stunting and anemia, still exist among low economic regions. This situation is referred to as the ‘double burden of malnutrition’ (DBM). DBM has become a serious problem in Southeast Asia. In a joint report with the World Health Organization (WHO) and the Association of Southeast Asian Nations, the United Nations Children’s Fund indicated that children from middle-income countries in Southeast Asia face the DBM [1]. In particular, Indonesians face serious health issues that arise from malnutrition. According to surveys conducted by the Indonesian government, the prevalence of anemia was 12.4% and 22.7% in male and female children aged between 13 and 18 years, respectively, while the prevalence of stunting was 35.1% and 31.4% in children aged between 13 and 15 years and between 16 and 18 years, respectively. Furthermore, the percentage of overweight individuals aged 16–18 years increased from 1.4% to 7.3% between 2010 and 2013 [1]. The government views nutritional problems among children as a top priority [2]. These nutritional problems are considered to be caused by the unfavorable dietary environment and behavior of Indonesian children. Most schools in Indonesia have not adopted a school meal program, and the implementation rate of the national system for the same was 0.14% in 2016 [3]. Consequently, children purchase from school stores and nearby stalls and consume unhealthy food items that are high in sugar and fat. This dietary habit is one of the causes of the DBM [4]. To address this issue, the Indonesian government has implemented a school feeding program in which supplementary food was provided. However, challenges arise due to allocation of resources, diversity within the country, and management issues, which resulted in low coverage of the program (0.14% in 2016) [3]. Hence, the impact of such programs towards students’ nutritional behavior and nutrition status was difficult to measure.

Since then, academic and private sectors have adopted similar meal programs to address other target groups, such as Islamic Boarding Schools. One of them was conducted by PT Ajinomoto together with IPB University. They conducted a School Lunch Program, which provided a balanced nutritious lunch combined with nutrition education.

This study aims to assess the impact of the school lunch program on the Knowledge, Attitude, and Practice (KAP) scores, dietary intake, nutritional status, and hemoglobin level of students. The result of this study is beneficial to determine the efficacy of the program and it is potential to be expanded in other educational institutions.

## 2. Materials and Methods

### 2.1. Research Design

A pre-post quasi-experimental study was conducted at one of the Islamic Boarding Schools in Java Island, West Java province, Indonesia. The intervention was a 9-month nutrition education program combined with the provision of a balanced lunch meal. Baseline measurements were taken in January and February 2018. The intervention was implemented between February and May and continued from July to December 2018. Midline and endline measurements were taken on the same items during the last half of each phase.

### 2.2. Participants

The participants of this study were male and female junior and senior high school students aged between 13 and 18 years who had attended the selected Islamic Boarding School for one or more years. An informed consent briefing was held in December 2017 for the parents or guardians of 450 potential participants. Students whose parents or guardians submitted written informed consent forms were included in this study. In total, 413 participants were screened for nutrition status and hemoglobin level. Students in their final year of school, having difficulty participating in the educational program, suffering from an infectious disease, having a blood hemoglobin (Hb) level of 7 g/dL or lower, and with a body mass index for age z-score (BAZ) of −3 or lower were excluded from the study. The excluded students also received dietary and educational interventions.

A subgroup of 115 students who were anemic and/or underweight and/or stunted was specifically investigated further for their nutritional intake, nutrition status, and hemoglobin level for the specific purpose of investigating the program’s impact before and after the intervention. As per the WHO guidelines, the definitions of anemia and underweight were male students with a blood Hb level of 12 g/dL or lower and female students with a blood Hb level of 11 g/dL or lower and students with a BMI-for-age z-score of −2 or lower, respectively. Stunting is defined as Height-for-age z-score of <−2 or lower [5]. The proportion of students who were anemic was 42% (*n* = 49), underweight 6% (*n* = 7), and stunted 70% (*n* = 80). Some students had multiple malnutrition, for example stunting and anemia, hence the total is not 100%. Sample size calculations were based on WHO recommendations [6]. The minimum sample size required for the subgroup analysis was based on the sample size calculated to detect a minimum change of 0.57 g/dL in the participants’ hemoglobin levels, with a standard deviation (SD) of 1.01 g/dL, a 95% confidence interval, and a statistical power of 0.80. This resulted in a minimum of 86 participants. A total of 115 participants were included to account for anticipated dropouts.

### 2.3. Intervention Methods

#### 2.3.1. Dietary Intervention

One meal per day was provided during lunchtime at the school. Recommended dietary allowances (RDAs) and recommended daily portions by age as defined for the Indonesian population were used [7,8]. In the present study, the following two criteria were selected for the nutrient content in school meals and ingredients to be used based on the average values for males and females aged between 13–15 and 16–18 years:

(a) 30% of RDA calories and proteins per school meal
  - Calories: 635–776 kcal/meal
  - Protein: 18–22 g/meal
(b) Includes staple food, animal-/plant-derived menus, vegetables, and fruits

Based on these conditions, 14 menu items were developed. School meals were provided in the dormitory cafeteria between 13:00 and 14:00 h six times a week. Details on meals provided are presented in Supplementary Table S1.

#### 2.3.2. Educational Intervention

This was conducted 2–3 times per month for a total of 25 sessions. One session was 15 to 45 min in duration and taught by the dormitory faculty; a research staff member was in charge once every three sessions. Twenty-five dormitory faculty members participated in training led by the research staff prior to the intervention. Two training sessions were conducted for each term for a total of four times. Meal caterers were educated on hygiene management and food safety once a month during the dietary intervention. Posters and banners were applied throughout the school to enhance the students’ knowledge, attitudes, and practices regarding health, nutrition, and hygiene.

### 2.4. Survey Items

#### 2.4.1. Primary Evaluation Items

- Knowledge, Attitude and Practice Test Scores

The KAP model is a commonly used approach for assessing knowledge, attitudes, and practices, particularly in the field of nutrition education. The model is based on the notion that attitudes are transformed by acquiring knowledge on nutrition and health and that practices are transformed owing to attitude transformation [9]. A KAP test designed for this study was used to assess the knowledge, attitudes, and practices of all students regarding health, nutrition, and hygiene. The questionnaire comprised 15 questions, including 11 questions on a balanced diet, 3 questions on hygiene, and 1 question on exercise. Knowledge-based questions could be answered by “yes” or “no” and questions on attitude by “agree” or “disagree”, with the score being the number of correct responses for both cases. Questions on practice were based on the frequency of behaviors on a scale of 5, and the score was calculated based on the responses (“always” = 4 points, “frequently” = 3 points, “sometimes” = 2 points, “rarely” = 1 point, and “never” = 0 point) (see Supplementary Materials).

- Blood hemoglobin level

Blood hemoglobin levels were measured using the HemoCue^®^ Hb 201 DM analyzer (Ängelholm, Sweden). The measurement was carried out at baseline, and the results were used to select students for the undernutrition subgroup. The Hb levels in the subgroup were measured for the midline and endline measurements.

#### 2.4.2. Secondary Evaluation Items

- Nutrition intake and nutrient adequacy ratio (NAR)

We estimated the intake of calories, proteins, fat, carbohydrates, iron, vitamin C, and vitamin A from daily meals and lunch using the 24 h recall method and by meal evaluation based on a semi-quantitative frequency questionnaire. Using the following formula, we also calculated the NAR for daily meals and lunches based on the male/female average values of RDA for those aged between 13–15 years and 16–18 years (Table 1).

NAR per day (%) = Daily nutritional intake/RDA

NAR during lunch (%) = Nutritional intake during lunch/1/3 RDA

- Nutrition status items

Body weight and height were measured to calculate the body mass index-for-age z-score. Weight was measured using a Camry body scale, and height was measured using a microtoise. Measurements were conducted by trained enumerators a minimum of twice. Z-score was calculated using the WHO A—315

Anthro-plus software and later classified based on the WHO Multicentre Growth Reference Study [10].

### 2.5. Statistical Analysis

Statistical analysis was performed using SPSS for Windows ver.20 (IBM, Armonk, NY, USA). The significance level was set at *p* < 0.05. Data from participants with missing values were excluded. To analyze the changes in each variable and compare the results of the baseline, midline, and endline measurements, the Friedman test was used.

### 2.6. Review and Approval by the Ethics Review Committee

This study was reviewed by the Ajinomoto Institutional Review Board of Ajinomoto Co., Inc. (Approval Number: 2017-025) and by the Ethics Review Committee of Bogor Agricultural University to ensure that ethical considerations were complied with (023/IT3. KEPMSM-IPB/SK/2018). This study was conducted in accordance with the Declaration of Helsinki.

## 3. Results

### 3.1. Baseline Results

Students whose data were collected for all evaluation items were analyzed. The total number of students was 319, of which 140 were males and 179 were females. The total number of students in the subsample was 115, of whom 35 were males and 80 were females. We collected questionnaires and all survey items from 73.5% of the students who submitted the study consent form. The characteristics of the analyzed participants are summarized in Table 1. The male-to-female ratio was approximately 4:6 for all students, but the percentage of female students was higher in the undernutrition subgroup. The male-to-female ratio in the undernutrition subgroup was 3:7. There was no significant difference between the students as a whole and within the subgroup in the number of students by grade level, and each grade level comprised 10% or more of the students in the study.

### 3.2. Results on the Health, Nutrition, and Hygiene Aspects of the Knowledge, Attitude, and Practice Test

Changes in the overall score for the health, nutrition, and hygiene items of the KAP test showed that all aspects significantly improved among the total students and undernutrition subgroup (Table 2). Both groups showed similar improvements. We subsequently examined the correct response rate for each question regarding knowledge and attitude (Table 3). The results showed a similar tendency between the total group of students and the subgroup in the correct response rate at the endline. Additionally, 12 out of the 15 questions on knowledge, and 14 out of the 15 questions on attitude had a correct response rate of over 70%. The scores on practice showed varied differences between the total student group and the subgroup at baseline, midline, and endline (Table 4). Therefore, we focused on improving practice frequency and categorized endline–baseline changes as improved and unimproved to observe the distribution (Table 5). The results showed that a higher percentage of participants in the subgroup demonstrated improved practice than the total group of students.

### 3.3. Blood Hemoglobin Levels and Anemia

**Figure 1:**
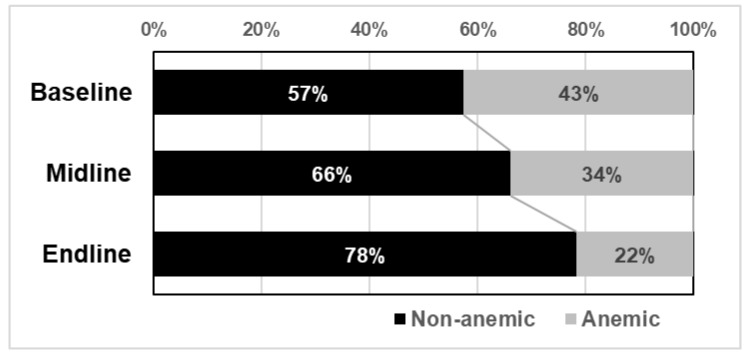
Changes of percentage of anemic and non-anemic students in the subgroup.

Table 6 shows changes in the blood Hb levels of the participants in the subgroup. Blood Hb levels were significantly improved at the time of endline compared to baseline and midline measurements. Furthermore, based on guidelines provided by WHO [5], the participants with a blood Hb level of 12 mg/dL or lower were categorized as anemic and those with a higher blood Hb level as non-anemic to observe the distribution (Figure 1). The percentage of anemic participants decreased by 21% from baseline to endline.

### 3.4. Nutritional Intake

Changes in nutritional intake from daily meals and lunch were analyzed using the dietary records of the students in the subgroup (Table 7). The results showed that the intake of all nutrients from lunch increased significantly. There was a significant increase in all nutrient contents except for total calories and carbohydrate contents. The NAR was calculated when 1/3 RDA was 100% (Table 8). Before the intervention, the adequacy ratio was below 50% for all nutrients. After the intervention, the adequacy ratio was >60% for energy, proteins, fat, and carbohydrates.

**Figure 2:**
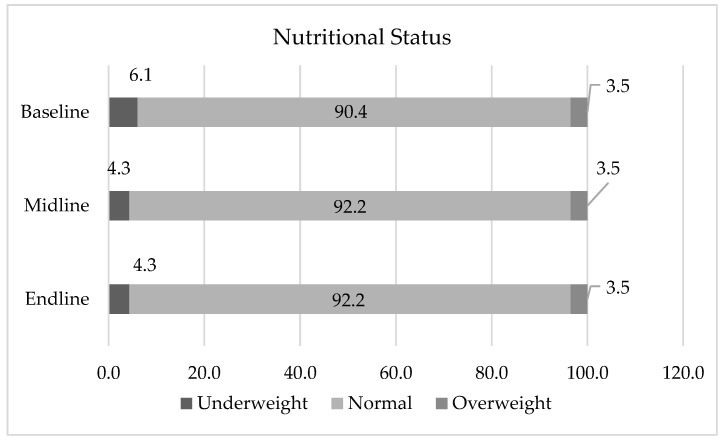
Changes in nutritional status of students.

Table 9 shows the change in BAZ of the students in baseline, midline, and endline. Meanwhile, Figure 2 shows the nutritional status of the students in baseline, midline, and endline divided into underweight (BAZ < −1), Normal (−1 < BAZ < 1), and overweight (BAZ > 1). The percentage of students with underweight nutritional status decreased (6.1% to 4.3% then 4.3%). This condition is similar to the normal status. Meanwhile, the percentage of overweight nutritional status shows no significant changes. The Friedman test shows that there was no difference in the nutritional status of students between the beginning, middle, and end of the program (*p* > 0.05). The condition of nutritional status of the students at the beginning of the program was quite good (94.5% of normal nutritional status) and remained good at the end of the program (95.1% of normal nutritional status).

### 3.5. Factors Affecting Blood Hemoglobin Levels

We performed a simple linear regression analysis with the rate of change in blood Hb levels (endline to baseline) as the dependent variable and the rates of change in the score of each practice item and in the adequacy ratio of each nutrient as explanatory variables to investigate factors that might have affected anemia improvement (Table 10). The energy and iron adequacy ratios and the rate of change in the practice score for protein consumption showed a positive correlation with changes in blood Hb levels. However, fat and carbohydrate adequacy ratios were negatively correlated with the rate of change in the practice score for physical activity.

## 4. Discussion

In the present study, nutrition and hygiene education and nutritionally balanced school lunches were provided to students in an Indonesian Islamic boarding school. We evaluated the impact of the intervention program by determining the KAP test scores, the values of biological indicators, nutritional intake, and other measurements.

The changes in the KAP scores for nutritional balance and hygiene were studied. The average knowledge score significantly increased after the intervention in the total student group. Upon analyzing the correct response rate, 12 out of the 15 questions accounted for over 70%. However, the correct response rates remained low after the intervention for “protein source”, “fiber consumption”, and “pyramid of a balanced diet” at 14.4%, 28.8%, and 42.9%, respectively. To explore which part of the educational intervention was most effective, we looked into the “balanced diet”, “physical activity”, and “handwashing” components, whichever showed a marked increase in the correct answer rate. These high-scoring components had an interactive program, such as card games and interview-type workshops. A participative approach is recommended as an effective mode of education for knowledge enhancement, not only in nutrition but also in a wide range of fields [11].

The overall average score on the attitude section increased significantly after the intervention. The correct response rate for attitude according to each item tended to be higher than that for knowledge, both during pre- and post-intervention. For example, the correct response rate at the endline for “fiber consumption” was 28.8% for knowledge (“constipation is caused by protein deficiency”), while it was 91.3% for attitude (“whether vegetable/fruit intake is necessary for preventing constipation”). In the KAP test, guidelines set forth by the Food and Agriculture Organization of the United Nations were followed. Knowledge was defined as understanding a specific topic, whereas attitude was defined as a positive or negative emotion/perception of a specific behavior [9]. Therefore, it is suggested that the perception of appropriate behavior regarding health and nutrition may not depend on having or lacking relevant knowledge.

Finally, the results of practice showed a significant improvement in the overall score after the intervention; however, the score was 39 out of 50 after the intervention, which was much lower than that of knowledge and attitude. This is assumed to be because questions on knowledge and attitude were two-choice questions, whereas questions on practice were regarding practice frequency and used a 5-point response scale, resulting in a smaller proportion of the total score on the test. Therefore, the students were divided in the subgroup into an improved group and a non-improved group based on the changes in practice frequency, and the data were compiled by item to investigate the improvement in practice in more detail. The results showed that items such as “anemia”, “pyramid of a balanced diet”, “protein source”, “dietary fiber consumption”, and “adolescent nutrition” improved in more than half of the participants. Xu et al. reported that knowledge enhancement improves attitudes and practices [12]. However, the results of the present study showed that the correct response rate after the intervention for knowledge and attitude was low for items for which marked improvement was seen in practice. There are two possible reasons for this result. The first is that questions of items on the same topic but with different terms in the KAP questionnaires resulted in the low percentage of correct responses. For example, for “anemia”, knowledge about anemia and iron intake was examined in the knowledge questionnaire and “action to be taken in an emergency to help an anemic person” was the phrase used in the attitude questionnaire and the “intake frequency of iron-containing foods” in the practice questionnaire. The questions for the attitude questionnaire differed from the other two, resulting in a non-linked improvement in scores compared with those of the knowledge and practice section of the KAP test. The second possible reason is the effect of the dietary intervention. The items that improved in the practice section were related to diet and nutrition. The increase in the practice frequency was maybe due to the results of the “school meals” that were provided in which the students had an opportunity for semi-forcibly practicing these items.

In terms of nutrition status, the study found no significant impact of the program on changes in the BMI-for-age z-score. This is different from the findings of a systematic review and meta-analysis done by Wang et al., who found that school feeding programs increased the weight and height of students after one year of intervention [13]. Hence, a longer duration of the SLP program could be suggested to result in changes in BAZ. To confirm the program’s effects on anemia status, we analyzed the distribution of the anemic and non-anemic groups before and after the intervention. We discovered that the percentage of those who were anemic had significantly decreased. This was most likely due to nutritional intervention, as the intake of iron and protein significantly increased in the subgroup. Simple linear regression analysis showed a positive correlation in the change in the blood Hb to the differences in the iron adequacy ratio and the practice score for “protein source”, respectively. Similar findings were observed in a school feeding program conducted by Sekiyama et al. [14] and Adelman et al. [15], but differed from Amani et al. [16], who discovered that nutrition education improves dietary practices but not hematologic indices. It can be assumed that total protein intake during the intervention period increased as the practice score for “protein source” increased, indicating an increase in the intake frequency of protein-source foods. Therefore, the increase in the iron adequacy ratio and total protein intake may have led to hemoglobin synthesis and improvement in anemia. In contrast, there was no correlation between the protein adequacy ratio calculated based on data from the 24 h dietary recall and blood Hb levels. These results suggest that an increase in the frequency of the protein intake from meals may be more important for anemia improvement than the amount of protein per meal.

The present study has two limitations. First, it is a pre-post study. As the trial was conducted at a single school, it was difficult to acquire a control group that did not undergo dietary and educational intervention owing to considerations regarding the conduction of the trial and ethics. To accurately demonstrate the intervention effects adequately, an intergroup comparison between the intervention and control groups is necessary. In addition, to see the single effect of dietary and educational interventions, two additional groups (educational and dietary) are needed. This issue should be addressed in future studies. Second, lunch was the only meal that was subjected to dietary intervention. Dinner skipping appeared as a new issue as a result of implementing dietary intervention for lunch only and educating students on the importance of breakfast. One reason is that making lunch more nutritionally balanced led to higher satisfaction. To improve the school meal program, these limitations must be overcome when designing the program.

## 5. Conclusions

The findings of this study suggest that a program combining dietary and educational interventions may effectively improve knowledge, attitudes, and practices regarding health, nutrition, and hygiene among junior and senior high school students. In addition, anemia improvement was observed in students with undernutrition.

## Figures and Tables

**Table 1:** Characteristics of the participants.

	Total Students		Subgroup		Subgroup/ Total Students
	(*n*)	(%)	(*n*)	(%)	(%)
Total	319	-	115	-	36.1%
Sex					
Male	140	43.9%	35	30.4%	25.0%
Female	179	56.1%	80	69.6%	44.7%
Grade					
1st	119	37.3%	33	28.7%	27.7%
2nd	50	15.7%	15	13.0%	30.0%
3rd	62	19.4%	19	16.5%	30.6%
4th	32	10.0%	14	12.2%	43.8%
5th	56	17.6%	34	29.6%	60.7%

**Table 2:** Scores of the KAP test in baseline, midline, and endline.

	Total Student (*n* = 319)				Subgroup (*n* = 115)			
	Median (25%, 75%)			*p*-Value	Median (25%, 75%)			*p*-Value
	Baseline	Midline	Endline	(Friedman)	Baseline	Midline	Endline	(Friedman)
Knowledge	9 (8–10)	12 (11–13)	12 (11–13)	<0.001	9 (8–10)	12 (10–13)	12 (11–13)	<0.001
Attitude	12 (11–13)	14 (12–14)	14 (13–15)	<0.001	12 (11–13)	14 (12–14)	14 (13–15)	<0.001
Practice	29 (25–33)	37 (33–42)	39 (34–43)	<0.001	29 (25–33)	37 (32–43)	39 (34–43)	<0.001

**Table 3:** The response rate of knowledge and attitude in the KAP test at baseline, midline, and endline.

			Total Student (*n* = 319)			Subgroup (*n* = 115)		
			Correct Response (%)			Correct Response (%)		
	Themes	Questions	Baseline	Midline	Endline	Baseline	Midline	Endline
Knowledge	Clean and Healthy Lifestyle	Dressing neatly and cutting nails are not one of the clean and healthy living habits.	92.2	94.7	94	88.7	94.8	93.0
	Drinking Water	We are recommended to drink five glasses of water every day.	91.2	78.7	87.1	93.0	72.2	87.0
	Breakfast	Breakfast is required as a major energy source before starting daily activities.	2.5	99.7	97.5	2.6	72.2	99.1
	Anemia	Anemia/lack of blood is due to not eating enough iron-rich foods.	89	89.3	98.1	89.6	87.0	98.3
	Vegetable Consumption	Teenagers who do not like vegetables tend to become obese in adulthood.	55.8	67.4	76.5	45.2	61.7	76.5
	Food Label	Food labels can provide information about the nutritional contribution of the food to our daily nutritional requirements.	81.8	92.5	91.2	77.4	92.2	91.3
	Salt, Sugar, and Fat	We don’t need to limit our sugar, salt, and fat consumption because they benefit our bodies.	97.5	94.4	96.2	98.3	93.0	94.8
	Balanced Diet	Indonesia has a balanced nutrition guide which consists of four pillars of balanced nutrition, which are: consuming diverse foods, doing physical activity, clean living habits, and weight monitoring.	53.9	96.9	91.5	56.5	95.7	95.7
	Pyramid of Balanced Diet	The balanced nutrition guidelines are illustrated in the form of a food pyramid; the group of foods containing carbohydrates is located at the top because we eat the most of these every day.	31.3	27.3	42.9	33.9	24.3	51.3
	Protein Source	Milk, eggs, chicken, meat, and beans are in the building nutrients group.	11	18.2	14.4	8.7	86.1	17.4
	Physical Activity	It is recommended to do physical activity or exercise at least once a week for 1 h.	32	64.9	72.1	30.4	67.0	73.0
	Fiber Consumption	Constipation is due to a lack of protein.	27.3	38.6	28.8	31.3	35.7	31.3
	Hand Washing	The right way to wash hands is using running water and soap.	34.8	99.4	99.7	47.0	99.1	100.0
	Adolescent Nutrition	A lack of nutrients in young women can cause malnutrition during pregnancy	85.3	88.4	96.2	86.1	87.0	98.3
	Surroundings Cleanness	If the surroundings are dirty and unhygienic, a person can easily contract diseases.	84.6	97.8	91.5	85.2	97.4	93.9
Attitude	Clean and Healthy Lifestyle	Dressing neatly and cutting my nails have no effect on my health	92.5	90.6	87.5	94.8	91.3	93.0
	Drinking Water	Drinking five glasses of water is enough to fulfill my requirements	78.7	80.9	85.9	80.9	71.3	86.1
	Breakfast	Breakfast is important to me, because otherwise I will have trouble concentrating in school	95.3	97.5	90.9	93.9	97.4	92.2
	Anemia	You shouldn’t worry if someone is tired, weak, lethargic, and pale	92.5	92.8	96.2	90.4	87.8	95.7
	Vegetable Consumption	I must consume vegetables every day to improve my digestion	49.8	97.2	90	49.6	96.5	94.8
	Food Label	I don’t consider nutrition and health information on food labels when choosing food	71.2	85	84.6	73.0	85.2	90.4
	Salt, Sugar, and Fat	I will choose foods with less sugar, salt, and fat even though these are not as tasty as foods high in sugar, salt, and fat	76.5	73	76.2	75.7	71.3	80.0
	Balanced Diet	I can implement the four pillars of balanced nutrition guidelines in my daily life	80.6	94.7	94	80.9	91.3	94.8
	Pyramid of Balanced Diet	The balanced nutrition pyramid helps me choose the right foods	85	96.9	93.4	83.5	94.8	94.8
	Protein Source	Eating tofu and tempeh alone is enough for building cells and tissues in our body	50.5	60.2	60.5	56.5	60.9	64.3
	Physical Activity	I need to do physical activity 5 times a day in a week, for at least 30 min	64.9	71.5	81.8	66.1	74.8	85.2
	Fiber Consumption	I need to consume vegetables and fruits to avoid constipation	96.2	95.6	91.2	93.9	97.4	91.3
	Hand Washing	Washing hands with running water is enough, if hands do not look dirty	70.2	89	76.8	76.5	90.4	78.3
	Adolescent Nutrition	I don’t need to worry about my nutritional status as a future parent now	89	90	88.1	88.7	87.0	90.4
	Surroundings Cleanness	I need to pay attention to the cleanliness of the surrounding environment because it will affect my health	91.2	98.1	98.4	93.0	100.0	98.3

**Table 4:** Scores on practice in the KAP test at baseline, midline, and endline.

Themes	Questions	Total Student (*n* = 319)				Subgroup (*n* = 115)			
		Median (25–75%)			*p*-Value (Friedman)	Median (25%, 75%)			*p*-Value (Friedman)
		Baseline	Midline	Endline		Baseline	Midline	Endline	
Clean and Healthy Lifestyle	How often are you neatly and cleanly dressed?	4 (3–4)	4 (4–4)	4 (4–4)	0.066	4 (4–4)	4 (4–4)	4 (4–4)	<0.05
Drinking Water	How often do you drink eight glasses of water per day?	3 (2–4)	3 (2–4)	3 (2–4)	<0.05	3 (2–4)	3 (2–4)	3 (2–4)	0.186
Breakfast	How often do you have breakfast before 9 o’clock?	4 (3–4)	4 (3–4)	4 (3–4)	<0.05	4 (3–4)	4 (3–4)	4 (3–4)	0.237
Anemia	How often do you consume a source of iron (red meat, chicken liver, iron tablets)?	1 (0–1)	1 (1–2)	2 (1–2)	0.063	1 (1–1)	1 (1–2)	2 (1–2)	<0.05
Vegetable Consumption	How often do you eat vegetables?	3 (2–4)	4 (3–4)	3 (3–4)	<0.05	3 (2–4)	4 (2–4)	3 (3–4)	<0.05
Food Label	Did you read the food label before deciding to buy packaged food?	1 (0–2)	1 (0–2)	1 (1–2)	<0.05	1 (1–1)	1 (1–2)	2 (1–2)	<0.05
Salt, Sugar, and Fat	How often do you drink sweet drinks?	2 (1–2)	2 (2–3)	2 (1–3)	0.460	2 (1–3)	2 (2–3)	2 (1–3)	<0.05
Balanced Diet	How often do you weigh yourself?	0 (0–1)	1 (1–1)	1 (1–1)	<0.05	1 (1–1)	1 (1–1)	1 (1–1)	<0.05
Pyramid of Balanced Diet	How often do you use the balanced food pyramid as a food guide?	0 (0–1)	2 (1–3)	2 (1–4)	<0.05	1 (1–1)	2 (1–3)	3 (1–4)	<0.05
Protein Sauce	How often do you consume sources of animal protein? (eggs, red meat, chicken)	1 (1–2)	2 (1–3)	2 (1–3)	<0.05	1 (1–2)	2 (1–3)	2 (1–3)	<0.05
Physical Activity	How often do you do physical activity continuously for at least 30 min?	2 (1–2)	2 (1–3)	2 (1–3)	<0.05	1 (1–2)	2 (1–3)	2 (1–3)	<0.05
Fiber Consumption	How often do you eat fruit?	1 (1–2)	3 (3–4)	4 (3–4)	<0.05	1 (1–2)	4 (3–4)	4 (3–4)	<0.05
Hand Washing	Did you wash your hands after using the bathroom?	3 (2–4)	4 (2–4)	4 (2–4)	<0.05	3 (2–4)	4 (2–4)	4 (3–4)	0.605
Adolescent Nutrition	How often do you seek nutrition and health information?	1 (0–1)	1 (1–2)	2 (1–2)	0.736	1 (1–1)	1 (1–2)	2 (1–3)	<0.05
Surroundings Cleanness	Do you help to clean up your neighborhood?	4 (3–4)	4 (3–4)	4 (3–4)	<0.05	4 (3–4)	4 (3–4)	4 (3–4)	0.251

**Table 5:** Percentage of improvement or non-improvement from baseline to endline on practice section in the KAP test.

Themes	Questions	Total Student (*n* = 319)				Subgroup (*n* = 115)			
		Improved		Not Improved		Improved		Not Improved	
		%	*n*	%	*n*	%	*n*	%	*n*
Clean and Healthy Lifestyle	How often are you neatly and cleanly dressed?	9.4%	30	69.9%	223	14.8%	17	82.6%	95
Drinking Water	How often do you drink eight glasses of water per day?	10.7%	34	66.1%	211	29.6%	34	66.1%	76
Breakfast	How often do you have breakfast before 9 o’clock?	5.0%	16	84.0%	268	14.8%	17	83.5%	96
Anemia	How often do you consume a source of iron (red meat, chicken liver, iron tablets)?	18.2%	58	42.3%	135	59.1%	68	32.2%	37
Vegetable Consumption	How often do you eat vegetables?	12.9%	41	58.9%	188	38.3%	44	55.7%	64
Food Label	Did you read the food label before deciding to buy packaged food?	15.4%	49	51.4%	164	43.5%	50	49.6%	57
Salt, Sugar, and Fat	How often do you drink sweet drinks?	10.7%	34	66.1%	211	14.8%	17	82.6%	95
Balanced Diet	How often do you weigh yourself?	14.1%	45	54.9%	175	33.9%	39	60.9%	70
Pyramid of Balanced Diet	How often do you use the balanced food pyramid as a food guide?	23.8%	76	23.5%	75	67.0%	77	22.6%	26
Protein Sauce	How often do you consume sources of animal protein? (eggs, red meat, chicken)	18.5%	59	41.1%	131	53.0%	61	39.1%	45
Physical Activity	How often do you do physical activity continuously for at least 30 min?	14.1%	45	55.2%	176	37.4%	43	56.5%	65
Fiber Consumption	How often do you eat fruit?	27.0%	86	13.8%	44	77.4%	89	11.3%	13
Hand Washing	Did you wash your hands after using the bathroom?	9.4%	30	69.6%	222	26.1%	30	70.4%	81
Adolescent Nutrition	How often do you seek nutrition and health information?	21.0%	67	33.2%	106	67.0%	77	23.5%	27
Surroundings Cleanness	Do you help to clean up your neighborhood?	8.5%	27	73.4%	234	18.3%	21	79.1%	91

**Table 6:** Changes in blood hemoglobin levels at baseline, midline, and endline in the subgroup.

	Subgroup (*n* = 115)			
	Median (25–75 Percentile)			*p*-Value
	Baseline	Midline	Endline	(Friedman)
Hb concentration	12.5 (11.1–14.0)	12.6 (11.5–13.4)	13.1 (12.2–14.2)	<0.005

**Table 7:** Average lunch or daily nutritional intake in baseline, midline, and endline (*n* = 115).

	Lunch				Daily			
Nutrients	Baseline	Midline	Endline	*p*-Value (Friedman)	Baseline	Midline	Endline	*p*-Value (Friedman)
Energy (kcal)	239 ± 171	387 ± 136	434 ± 137.8	<0.05	1486 ± 720	1505 ± 498	1632 ± 489	0.12
Proteins (g)	7.5 ± 6.2	11.8 ± 5.5	11.1 ± 4.7	<0.05	19.0 ± 11	35.6 ± 14.3	36.3 ± 14.3	<0.05
Fat (g)	6.6 ± 7.8	12.1 ± 5.3	16.0 ± 5.9	<0.05	13.4 ± 8.6	21.8 ± 9.7	22 ± 11.2	<0.05
Carbohydrates (g)	37.8 ± 27.7	58.0 ± 24.4	62.0 ± 23.7	<0.05	219.8 ± 105.4	226.9 ± 77.2	238.2 ± 71.6	0.26
Iron (mg)	1.0 ± 0.9	2.1 ± 1.	2.7 ± 1.3	<0.05	2.7 ± 1	6.9 ± 0.9	7.0 ± 1.0	<0.05
Vitamin C (mg)	3.4 ± 4.3	30.9 ± 28.1	17.6 ± 15.0	<0.05	26.22 ± 39.4	55.8 ± 54.4	40.8 ± 44.1	<0.05

**Table 8:** Median NAR of students at baseline, midline, and endline during lunch (*n* = 115).

	Median (25–75 Percentile)			
Nutrients	Baseline	Midline	Endline	*p*-Value (Friedman)
Energy	44.2 (27.3–58.4)	61.7 (52.9–74.9)	68.5 (55.6–85.9)	<0.05
Proteins	45.1 (28.8–67.0)	65.5 (52.9–80.6)	59.1 (44.5–72.5)	<0.05
Fat	33.3 (48.3–60.4)	63.8 (43.5–75.8)	76.5 (60.4–93.2)	<0.05
Carbohydrates	43.7 (27.9–69.1)	67.0 (54.1–87.8)	71.3 (56.0–91.0)	<0.05
Fe	14.6 (8.0–24.0)	30.9 (19.9–35.0)	38.3 (26.7–45.9)	<0.05

**Table 9:** Changes in BAZ at baseline, midline, and endline of students.

	Subgroup (*n* = 115)			
	Median (25–75 Percentile)			*p*-Value
	Baseline	Midline	Endline	(Friedman)
BAZ	−0.09 (−0.7–0.8)	0.04 (−0.7–0.94)	−0.08 (−0.79–0.92)	0.607

**Table 10:** Single correlation analysis with the rate of change in blood Hb levels as the variable.

Variable	β Coef	*p*-Value	95% CI	
			Lower Limit	Upper Limit
Delta Nutrient Adequacy during Lunch				
Energy	5.292	0.04	0.054	2.261
Proteins	−0.642	0.132	−0.258	0.035
Fat	−2.774	0.03	−0.666	−0.034
Carbohydrates	−3.808	0.036	−1.275	−0.044
Iron	0.334	0.009	0.021	0.143
Vitamin C	−0.21	0.032	−0.028	−0.001
Delta Score KAP				
Knowledge	0.221	0.039	0.002	0.055
Attitude	0.024	0.822	−0.019	0.024
Practice	−0.023	0.826	−0.032	0.025
1. CHLB	0.092	0.388	−0.2	0.508
2. Drinking water	0.034	0.763	−0.262	0.355
3. Breakfast	−0.195	0.075	−0.52	0.025
4. Anemia	0.044	0.68	−0.269	0.411
5. Vegetable consumption	−0.006	0.955	−0.292	0.276
6. Food labels	0.039	0.676	−0.196	0.301
7. Sugar, salt, fat	−0.112	0.265	−0.378	0.106
8. Balanced diet	0.033	0.731	−0.325	0.461
9. Pyramid of balanced diet	−0.016	0.883	−0.235	0.202
10. Protein source consumption	0.267	0.007	0.109	0.652
11. Physical activity	−0.277	0.005	−0.561	−0.107
12. Fiber consumption	0.145	0.151	−0.075	0.474
13. Hand washing	−0.139	0.191	−0.445	0.091
14. Adolescent nutrition	−0.087	0.425	−0.381	0.163
15. Environmental cleanliness	−0.062	0.594	−0.429	0.248

## Data Availability

The data presented in this study are available on request from the corresponding author. The data are not publicly available owing to ethical reasons.

## Supplementary Materials

The following supporting information can be downloaded at: https://www.mdpi.com/article/10.3390/nu15041055/s1, Table S1: School Lunch Meal Plan of 14 Menu (Cycles).
