# Supplementary material for: School Lunch Programs and Nutritional Education Improve Knowledge, Attitudes, and Practices and Reduce the Prevalence of Anemia: A Pre-Post Intervention Study in an Indonesian Islamic Boarding School
Source: Nutrients. 2023 Feb 20;15(4):1055. doi: 10.3390/nu15041055 (PMC9962024; doi:10.3390/nu15041055)
Supplement: Supplementary file 1 [file nutrients-15-01055-s001.zip › nutrients-2212609-supplementary.pdf]

Supplementary Materials

Table S1. School Lunch Meal Plan of 14 Menu (Cycles).

Cycle	Breakfast	Lunch	Dinner
1	• Steamed rice<br>• Boiled vegetables and fried tofu with peanut sauce (P*ecel*)	• Steamed rice<br>• Fried mackerel<br>• Boiled mix vegetables wih grated coconut (*Urap*)<br>• Banana	• Steamed rice<br>• Tofu and oyster mushoom soup
2	• Steamed rice<br>• Mix long bean and tempeh stir fry	• Steamed rice<br>• Fried tofu with sweet and sour sauce<br>• Sprouts stir fry with mushroom<br>• Papaya	• Steamed rice<br>• Vegetable and tempeh cooked with coconut milk (*Lodeh*)
3	• Steamed rice<br>• Mix green bean and tofu stir fry	• Steamed rice<br>• Fried eggs<br>• Boiled Mix vegetables with peanut sauce (*Pecel*)<br>• Snake fruit	• Steamed rice<br>• Fried tofu<br>• Chayote stir fry with corn
4	• Steamed rice<br>• Vegetable and tempeh cooked with coconut milk (*Lodeh*)	• Steamed rice<br>• Fried tempeh coated with wheat flour (*Tempe mendoan*)<br>• Mix vegetables in tamarind soup (*Sayur asem*)<br>• Orange	• Steamed rice<br>• Mix vegetables soup
5	• Steamed rice<br>• Fried tempeh<br>• Pak coi stir fry	• Steamed rice<br>• Fried chicken<br>• Cassava leave cooked with coconut milk (*Gulai daun singkong*)<br>• Water melon	• Steamed rice<br>• Tempeh cooked with palm sugar sauce (*Tempe bacem*)<br>• Water Spinach (*Kangkung*) stir fry
6	• Fried rice (made from steamed rice, vegetable and seasoning)<br>• Fried egg	• Steamed rice<br>• Chicken liver cooked with chili sauce and coconut milk (*Rendang hati ayam*)<br>• Spinach soup with corn<br>• Melon	• Steamed rice<br>• Chayote and tempeh with curry soup
7	• Steamed rice<br>• Stir fry tempeh with soy sauce<br>• Chinese cabbage stir fry	• Steamed rice<br>• Mix green bean and tofu stir fry<br>• Banana	• Steamed rice<br>• Fried tofu<br>• Spinach soup
8	• Steamed rice<br>• Tofu and oyster mushroom soup	• Steamed rice<br>• Mackerel with chili sauce (*Tongkol balado*)<br>• Vegetable and tempeh cooked with coconut milk (*Lodeh*)<br>• Mangosteen	• Steamed rice<br>• Fried tempeh<br>• Mix vegetables in tamarind soup (*Sayur asem*)
9	• Steamed rice	• Steamed rice	• Steamed rice

	• Fried tempeh<br>• Mix vegetable stir fry (*Capcay*)	• Chicken soup with vegetable<br>• Rambutan	• Chayote and boiled egg with curry soup
10	• Steamed rice<br>• Fried tofu<br>• Pak coi stir fry	• Steamed rice<br>• Fried eggs with sweet and sour sauce<br>• Water Spinach (*Kangkung*) stir fry<br>• Orange	• Steamed rice<br>• Fried tempeh<br>• Oyster mushoom soup with carrot
11	• Steamed rice<br>• Fried tempeh<br>• Jack fruit cooked with coconut milk (*Gulai nangka*)	• Steamed rice<br>• Tempeh cooked with palm sugar sauce (*Tempe bacem*)<br>• Chinese okra/Luffa soup<br>• Papaya	• Steamed rice<br>• Fried Tofu<br>• Mix vegetables in tamarind soup (*Sayur asem*)
12	• Steamed rice<br>• Sprouts stir fry with tofu	• Steamed rice<br>• Fried chicken with oyster sauce<br>• Mix oyster mushroom and corn stir fry<br>• Snake fruit	• Steamed rice<br>• Fried Tempeh<br>• Chinese cabbage stir fry
13	• Steamed rice<br>• Vegetable and tempeh cooked with coconut milk (*Lodeh*)	• Steamed rice<br>• Chicken liver cooked with soy sauce (*Semur hati ayam*)<br>• Mix vegetable stir fry (*Capcay*)<br>• Banana	• Steamed rice<br>• Chayote and tofu with curry soup
14	• Steamed rice<br>• Pak coi stir fry with tofu	• Steamed rice<br>• Tempeh cooked with coconut milk (*Opor tempe*)<br>• Green bean stir fry<br>• Orange	• Steamed rice<br>• Fried tempeh<br>• Spinach soup with corn
